# Sensitivities in protein allocation models reveal distribution of metabolic capacity and flux control

**DOI:** 10.1093/bioinformatics/btae691

**Published:** 2024-11-18

**Authors:** Samira van den Bogaard, Pedro A Saa, Tobias B Alter

**Affiliations:** Institute of Applied Microbiology, Aachen Biology and Biotechnology, RWTH Aachen University, Aachen 52074, Germany; Departamento de Ingeniería Química y Bioprocesos, Escuela de Ingeniería, Pontificia Universidad Católica de Chile, Santiago 7820436, Chile; Instituto de Ingeniería Matemática y Computacional, Pontificia Universidad Católica de Chile, Santiago 7820436, Chile; Institute of Applied Microbiology, Aachen Biology and Biotechnology, RWTH Aachen University, Aachen 52074, Germany

## Abstract

**Motivation:**

Expanding on constraint-based metabolic models, protein allocation models (PAMs) enhance flux predictions by accounting for protein resource allocation in cellular metabolism. Yet, to this date, there are no dedicated methods for analyzing and understanding the growth-limiting factors in simulated phenotypes in PAMs.

**Results:**

Here, we introduce a systematic framework for identifying the most sensitive enzyme concentrations (sEnz) in PAMs. The framework exploits the primal and dual formulations of these models to derive sensitivity coefficients based on relations between variables, constraints, and the objective function. This approach enhances our understanding of the growth-limiting factors of metabolic phenotypes under specific environmental or genetic conditions. Compared to other traditional methods for calculating sensitivities, sEnz requires substantially less computation time and facilitates more intuitive comparison and analysis of sensitivities. The sensitivities calculated by sEnz cover enzymes, reactions and protein sectors, enabling a holistic overview of the factors influencing metabolism. When applied to an *Escherichia coli* PAM, sEnz revealed major pathways and enzymes driving overflow metabolism. Overall, sEnz offers a computational efficient framework for understanding PAM predictions and unraveling the factors governing a particular metabolic phenotype.

**Availability and implementation:**

sEnz is implemented in the modular toolbox for the generation and analysis of PAMs in Python (PAModelpy; v.0.0.3.3), available on Pypi (https://pypi.org/project/PAModelpy/). The source code together with all other python scripts and notebooks are available on GitHub (https://github.com/iAMB-RWTH-Aachen/PAModelpy).

## 1 Introduction

Genome-scale metabolic models (GSMMs) have proven valuable in improving our understanding of microorganisms and exploring their phenotypic behavior (Orth *et al.* 2010b). These constraint-based models rely on a stoichiometric representation of the biochemical network describing mass balances, in conjunction with a biologically relevant objective function and capacity constraints (Schuetz *et al.* 2007, Orth *et al.* 2010b). While GSMMs have enabled a diversity of analyses, they are often unable to capture more complex metabolic phenomena, such as overflow metabolism in bacteria like *Escherichia coli,* or the Crabtree effect in yeast, unless additional assumptions are made such as limited oxygen uptake (O’Brien *et al.* 2015, Sánchez *et al.* 2017). Prediction fidelity can be improved by including constraints related to macromolecular component limitations, thermodynamics, and enzyme kinetics, among others (Salvy and Hatzimanikatis 2020, Kerkhoven 2022). Various studies have focused on incorporating kinetic relations between reaction fluxes and the corresponding enzymes, thereby improving flux predictions (O’Brien *et al.* 2015, Sánchez *et al.* 2017, Alter *et al.* 2021) and recapitulating known phenomena like overflow metabolism (Beg *et al.* 2007, de Groot *et al.* 2020). For instance, the protein allocation model (PAM) of *E. coli* constrains the amount of proteins available for metabolic reactions (i.e. enzymes) by adding additional protein partitions associated to idle proteins and translation-related processes (Alter *et al.* 2021). While PAMs have enabled more accurate predictions than traditional GSMMs, mathematical methods for appropriately interpreting flux sensitivities and metabolic limitations have lagged behind.

Understanding optimization results requires insights into the relation between variables, constraints, and the objective function. By computing the sensitivity of the objective function to changes in both enzymes and fluxes (variables), as well as protein and flux bounds (represented by inequality constraints), insights can be gained into which metabolic processes influence the predicted phenotype. For kinetic models based on ordinary differential equations (ODEs), metabolic control analysis (MCA) theory offers a framework for calculating flux and concentration sensitivities on a systems level (Kacser and Burns 1973, Moreno-Sánchez *et al.* 2008). For constraint-based models, analogous sensitivities have been determined through perturbation of individual kinetic parameters and assessing the effect on the objective reaction flux, often the biomass production rate. However, computational costs of these algorithms are considerably high (Lu *et al.* 2019, Tsouka *et al.* 2021, Fan *et al.* 2023). Wilken *et al.* (2022) introduced a novel, computationally efficient approach based on differentiable constraint-based models for assessing the effect of kinetic parameter alterations. Nevertheless, this method offers little insights into reactions without associated parameters or broader growth-restricting processes. Moreover, to the best of our knowledge, this methodology has not been extended to PAMs.

Here, we introduce sEnz, a systematic and computationally efficient framework to study the metabolic limitations and most sensitive enzymes in PAMs. Our method uses the primal and dual formulation of these models to derive general mathematical relationships between variables, constraints, and the objective function value, describing the sensitivity of the latter to the total proteome, enzyme concentrations, and flux capacities. The sensitivities are described by normalized dual variables (shadow prices) encompassing: (i) capacity sensitivity coefficients (CSCs), sensitivities of active capacity constraints (fluxes, enzymes, and total proteome) limiting the objective function value, and (ii) enzyme sensitivity coefficients (ESCs), sensitivities that are related to the influence of individual enzyme concentration constraints on active capacity constraints (CSCs), i.e. the effect of individual enzymes on the constraint limiting the total protein content. We applied sEnz to different PAMs aiding in the interpretation of limitations of the growth rate in *E. coli* under varying degrees of glucose limitation, as well as under protein overexpression scenarios. sEnz is reminiscent of metabolic control analysis, offering a sound mathematical framework for interrogating metabolic limitations in PAMs.

## 2 Approach

GSMMs are mathematically described by the stoichiometric matrix ***S***${}_{m\times n}$, which encodes the mass balances for *m* metabolites reacting in *n* biochemical reactions with flux values represented by the flux vector **v** (Orth *et al.* 2010b). These reactions are thermodynamically and enzymatically limited by capacity constraints in the form of lower $v_{\text{min}}$ and upper $v_{\text{max}}$ bounds. Without loss of generality, **v** is constituted by non-negative flux values as each reversible reaction can be split into two non-negative, irreversible reactions in opposing directions. As a consequence, the number of reaction variables *n* and the length of **v** are twice the number of actual biochemical reactions represented in a typical GSMM. This necessarily implies that the flux bounds are equal to or greater than zero. The general flux balance analysis (FBA) formulation (P1) can be used to compute an optimal flux vector $\bar{v}$ that complies with mass balances and capacity constraints while maximizing an objective reaction flux *v_z_* encoded in the entries of the objective vector **c** (Fell and Small 1986, Orth *et al.* 2010b),

(1) $$\begin{array}{c} (P1)\;\max\;\;v_{z}=c^{T}v\\ s.t.\;S\cdot v=0\\ v_{\text{min}}\leq v\leq v_{\text{max}}\\ v_{i}\in R_{0}^{+}\\ i\in \{1,..,n\} \end{array}$$

PAMs expand the predictive capabilities of (P1) by incorporating three protein sectors representing protein partitions into the metabolic network. These sectors describe active enzymes ($\phi_{\text{AE}}$), unused enzymes ($\phi_{\text{UE}}$), and translation-related ($\phi_{T}$) protein concentrations, whose combined sum cannot exceed the total protein content per gram of biomass. The unused and translation-related protein sector are coarse-grained sectors that are linearly related to the substrate uptake rate and the growth rate, respectively. The active enzyme sector includes all enzymes related to metabolic reactions. The concentration of each enzyme is calculated by assuming that the reaction flux is proportional to the enzyme concentration, i.e. $e_{j}=v_{j}/k_{\text{cat},j}$, where $k_{\text{cat},j}$ denotes the catalytic constant in 1/h for enzyme *j*, and *e_j_* is its corresponding enzyme concentration in mmol/$g_{\text{CDW}}$. The catalytic constants can be assumed to represent the maximal ’*in vivo*’ catalytic efficiency of an enzyme, as the unused protein sector corrects for potential enzymes which are not used at their full capacity (Alter *et al.* 2021). Since intracellular space and resources are finite, the limited protein budget must be dynamically allocated to different functions or sectors. The allocation of the total protein budget $\phi_{\text{total}}$ in ${\text{mg}}_{\text{protein}/}{\text{g}}_{\mathrm{CDW}}$ to the protein sectors is defined by the following linear equations (Alter *et al.* 2021):

(2) $$\begin{array}{cc} & \phi_{\text{total}}\geq \phi_{\text{AE}}+\phi_{\text{UE}}+\phi_{T}\\ & \phi_{T}=\phi_{T,0}+w_{T}\cdot \mu \\ & \phi_{\text{UE}}=\phi_{\text{UE},0}-w_{\text{UE}}\cdot v_{s}\\ & \phi_{\text{AE}}=\sum_{j}^{E}m_{j}e_{j} \end{array}$$

where *m_j_* is the molar mass of enzyme *j* in g/mol, and $\phi_{T,0}$ and $\phi_{\text{UE},0}$ are the protein fractions at zero growth in ${\text{mg}}_{\text{protein}}/g_{\text{CDW}}$ of the translational and unused protein sector, respectively. $w_{T}$ and $w_{\text{UE}}$ represent the slopes of the relations between the translational protein sector and the growth rate *μ* in 1/h, and the unused enzyme sector and the substrate uptake rate $v_{s}$ in $\text{mmol}/g_{\text{CDW}}/h$, respectively. By inspecting Equation (2), the total protein constraint [line 1 in Equation (2)] can be rewritten as

(3) $$m^{T}e+w^{T}v\leq \phi_{0}$$

where $\phi_{0}=\phi_{\mathrm{total}}-\phi_{T,0}-\phi_{\text{UE},0}$ and $m^{T}e=\sum_{j}^{E}m_{j}e_{j}$. The weight vector **w** is only nonzero for the biomass equation and the substrate uptake rate, representing $w_{T}$ and $-w_{\text{UE}}$, respectively. **m** is a vector containing the molar masses of the $n_{\text{enz}}$ metabolic enzymes in g/mol. These resource allocation constraints can be appended to (P1), yielding the general PAM formulation (P2) (Alter *et al.* 2021),

(4) $$\begin{array}{c} (P2)\;\max\;\;v_{z}=c^{T}v\\ s.t.\;S\cdot v=0\\ K_{\text{inv}}\cdot v\leq e\\ w^{T}v+m^{T}e\leq \phi_{0}\\ v_{\text{min}}\leq v\leq v_{\text{max}}\\ e_{\text{min}}\leq e\leq e_{\text{max}}\\ v_{i},e_{j}\in R_{0}^{+}\\ i\in \{1,..,n\},\;j\in \{1,..,n_{\text{enz}}\} \end{array}$$

where $K_{\text{inv}}$ is a diagonal matrix with nonzero elements equal to the reciprocals of catalytic constants $k_{\text{cat}}$ in 1/h for $n_{\text{enz}}$ metabolic enzymes. $e_{\text{min}}$ and $e_{\text{max}}$ are lower and upper bounds on the enzyme concentrations in $\mathrm{mmol}/g_{CDW}$, respectively. A list of all the mathematical symbols and their units is provided in Supplementary Table S1.

### 2.1 Sensitivity coefficients from the dual formulation

The dual (D2) of the PAM formulation (P2) is given as follows (refer to Supplementary Text S1 for details):

(5) $$\begin{array}{c} (D2)\;\min\;v_{\text{max}}^{T}\mu_{\text{max}}-v_{\text{min}}^{T}\mu_{\text{min}}+e_{\text{max}}^{T}\varepsilon_{\text{max}}-e_{\text{min}}^{T}\varepsilon_{\text{min}}+\phi_{0}\pi \\ s.t.\;S^{T}\lambda +K_{\text{inv}}^{T}\xi +\mu_{\text{max}}-\mu_{\text{min}}+\pi \cdot w\geq c\\ -\xi +\varepsilon_{\text{max}}-\varepsilon_{\text{min}}+\pi \cdot m\geq 0\\ \pi ,\xi_{j},\mu_{\text{max},i},\mu_{\text{min},i},\varepsilon_{\text{max},j},\varepsilon_{\text{min},j}\in R_{0}^{+};\;\lambda_{i}\in R\\ i\in \{1,..,n\},\;j\in \{1,..,n_{\text{enz}}\} \end{array}$$

where $\left(\lambda ,\xi ,\mu_{\text{max}},\mu_{\text{min}},\varepsilon_{\text{max}},\varepsilon_{\text{min}},\pi \right)$ denote dual variables or shadow prices describing the sensitivities of the objective function value of (P2) upon changes on the right-hand side of the constraints. These variables can also be interpreted as unscaled response (sensitivity) coefficients. More importantly, if $\left(\bar{v},\bar{e}\right)$ and $\left(\bar{\lambda },\bar{\xi },{\bar{\mu }}_{\text{max}},{\bar{\mu }}_{\text{min}},{\bar{\varepsilon }}_{\text{max}},{\bar{\varepsilon }}_{\text{min}},\bar{\pi }\right)$ are respectively optimal solutions of the primal (P2) and the associated dual (D2) problem, then, by strong duality, the objective function values must be equal provided they are both feasible and finite. This result, along with an analysis of the constraints in (D2), provides an exact and efficient method for computing sensitivity relationships in PAMs.

### 2.2 Capacity sensitivities: definitions and capacity summation theorem

At the optimum, the objective function values of (P2) and (D2) fulfill,

(6) $${\bar{v}}_{z}=v_{\text{max}}^{T}{\bar{\mu }}_{\text{max}}-v_{\text{min}}^{T}{\bar{\mu }}_{\text{min}}+e_{\text{max}}^{T}{\bar{\varepsilon }}_{\text{max}}-e_{\text{min}}^{T}{\bar{\varepsilon }}_{\text{min}}+\phi_{0}\bar{\pi }$$

Upon normalization by a valid nonzero ${\bar{v}}_{z}$, a familiar summation result emerges,

(7) $$\begin{array}{c} \sum_{i=1}^{n}C_{\mu ,i}^{v_{z}}+\sum_{j=1}^{n_{\text{enz}}}C_{\varepsilon ,j}^{v_{z}}+C_{\pi }^{v_{z}}=1 \end{array}$$

where the above coefficients $C_{\mu ,i}^{v_{z}},\;C_{\varepsilon ,j}^{v_{z}}$, and $C_{\pi }^{v_{z}}$ represent capacity sensitivity coefficients (CSCs) that separately quantify the relative contribution of flux, enzyme, and proteome capacity to an increase in the objective function value ${\bar{v}}_{z}$ (Table 1). Alternatively, these coefficients reveal active constraints that limit the objective function (often biomass production). Note that a flux CSC corresponds to flux constraints (line 5 in (P2)), enzyme CSC to enzyme capacity constraints (line 6 in (P2)), and proteome CSC to the total protein constraint (line 4 in (P2)) (refer to Table 1).

**Table 1.** Sensitivity coefficients and parameters definitions used in this study, along with the associated dual variables^a^

Description	Mathematical definitions	Dual variables
**Sensitivities**		
Flux capacity sensitivity coefficient (flux CSC)	$C_{\mu ,i}^{v_{z}}=(1/{\bar{v}}_{z})(v_{\text{max},i}\cdot {\bar{\mu }}_{\text{max},i}-v_{\text{min},i}\cdot {\bar{\mu }}_{\text{min},i})=({\bar{v}}_{i}/{\bar{v}}_{z})\Delta {\bar{\mu }}_{i}$	$\mu_{\text{min},i},\;\mu_{\text{max},i}$ , flux bounds
Enzyme capacity sensitivity coefficient (enzyme CSC)	$C_{\varepsilon ,i}^{v_{z}}=(1/{\bar{v}}_{z})(e_{\text{max},j}\cdot {\bar{\varepsilon }}_{\text{max},j}-e_{\text{min},j}\cdot {\bar{\varepsilon }}_{\text{min},j})=({\bar{e}}_{j}/{\bar{v}}_{z})\Delta {\bar{\varepsilon }}_{j}$	$\varepsilon_{\text{min},i}$ , $\varepsilon_{\text{max},i}$ enzyme concentration bounds
Proteome capacity sensitivity coefficients (proteome CSC)	$C_{\pi }^{v_{z}}=(\phi_{0}/{\bar{v}}_{z})\bar{\pi }$	*π*, total protein constraint
Enzyme sensitivity coefficient (ESC)	$C_{e,j}^{v_{z}}=({\bar{e}}_{j}/{\bar{v}}_{z}){\bar{\xi }}_{j}$	*ξ_j_*, enzyme constraints
**Parameters**		
Active enzyme sector (AES)	$\alpha =(1/\phi_{0})\sum_{j=1}^{n_{\text{enz}}}{\bar{e}}_{j}$	–

a Parameter indices (*i*, *j*) denote a single reaction and enzyme, respectively. Decision variables correspond to the optimal solutions found for (P2) and (D2). The description and units of all the mathematical symbols can be found in Supplementary Table S1.

### 2.3 Enzyme sensitivities: definitions and connectivity relationships

The constraints in (D2) provide relationships between primal and dual variables at any feasible tuple ($\hat{v},\;\hat{e},\;\hat{\lambda },\;\hat{\xi },\;{\hat{\mu }}_{\text{max}},\;{\hat{\mu }}_{\text{min}},\;{\hat{\varepsilon }}_{\text{max}},\;{\hat{\varepsilon }}_{\text{min}},\;\hat{\pi }$). Left multiplication of the first and second constraints in (D2) by ${\hat{v}}^{T}$ and ${\hat{e}}^{T}$ gives respectively,

(8) $${\hat{v}}^{T}\left[K_{\text{inv}}^{T}\hat{\xi }+{\hat{\mu }}_{\text{max}}-{\hat{\mu }}_{\text{min}}+\hat{\pi }\cdot w\right]\geq {\hat{v}}_{z}$$

(9) $${\hat{e}}^{T}\left[-\hat{\xi }+{\hat{\varepsilon }}_{\text{max}}-{\hat{\varepsilon }}_{\text{min}}+\hat{\pi }\cdot m\right]\geq 0$$

where the result ${\hat{v}}^{T}S^{T}={\left(S\hat{v}\right)}^{T}=0$ is used. Analysis of (P2) reveals that: (i) the modeled proteome is always fully allocated to the protein sectors, so that the total proteome constraint is active, (ii) ${\hat{e}}^{T}={\left(K_{\text{inv}}\hat{v}\right)}^{T}$ as a consequence of (i), and (iii) the shadow price associated to a flux capacity constraint (${\hat{\mu }}_{i}$) is nonzero only if ${\hat{v}}_{i}=v_{\text{max},i}$ or ${\hat{v}}_{i}=v_{\text{min},i}$, i.e. ${\left({\hat{v}}_{i}{\hat{\mu }}_{i}\right)}_{\text{max}}-{\left({\hat{v}}_{i}{\hat{\mu }}_{i}\right)}_{\text{min}}={\hat{v}}_{i}\Delta {\hat{\mu }}_{i}$. Application of the previous relations followed by summation of Equations (8) and (9) using ${\hat{v}}^{T}w+{\hat{e}}^{T}m=\phi_{0}$ from (i) yields,

(10) $$v_{\text{max}}^{T}{\hat{\mu }}_{\text{max}}-v_{\text{min}}^{T}{\hat{\mu }}_{\text{min}}+e_{\text{max}}^{T}{\hat{\varepsilon }}_{\text{max}}-e_{\text{min}}^{T}{\hat{\varepsilon }}_{\text{min}}+\phi_{0}\hat{\pi }\geq {\hat{v}}_{z}$$

Notably, the above inequality becomes an equality at the optimum as per strong duality, and thus, also Equations (8) and (9) become equalities. Then, at the optimum and upon normalization of each summation by a valid nonzero ${\bar{v}}_{z}$, the following connectivity relationships are found from the latter equations (refer to Table 1 for notation).

(11) $$\sum_{j=1}^{n_{\text{enz}}}C_{e,j}^{v_{z}}+\sum_{i=1}^{n}C_{\mu ,i}^{v_{z}}+(1-\alpha )\cdot C_{\pi }^{v_{z}}=1$$

(12) $$-\sum_{j=1}^{n_{\text{enz}}}C_{e,j}^{v_{z}}+\sum_{j=1}^{n_{\text{enz}}}C_{\varepsilon ,j}^{v_{z}}+\alpha \cdot C_{\pi }^{v_{z}}=0$$

where $C_{e,j}^{v_{z}}$ represents the enzyme sensitivity coefficient (ESC) of reaction *z* for enzyme *j*, and *α* denotes the proteome fraction allocated to metabolic enzymes or active enzyme sector (AES) (Table 1). An ESC can be understood as the fractional increase of the objective function value upon the increase of an enzyme concentration at the optimum ${\bar{v}}_{z}$. In contrast to the enzyme CSC, which relates to the enzyme concentration bounds (line 6 in (P2)), the ESC relates to the enzyme concentration variables in the PAM as depicted in Table 1 (line 3 in (P2)). Notably, this quantity is non-negative by construction [refer to (P2) and Table 1]. Inspection of Equation (12) reveals that the sum of ESCs is nonzero only if either the proteome CSC ($C_{\pi }^{v_{z}}$) is nonzero (i.e. the total enzyme pool is limiting) and/or at least one enzyme CSC ($C_{\varepsilon ,j}^{v_{z}}$) is nonzero (i.e. at least one enzyme is at its highest concentration). In such cases, at least one nonzero ESC indicates how relative changes in the corresponding enzyme concentration affects the relative objective function value. If the proteome and all enzyme CSCs are zero, then the ESCs sum is zero, and consequently, the model predictions are only flux-capacity limited, i.e., the model becomes a conventional GSMM. The latter property distinguishes ESCs from traditional flux control coefficients, as these coefficients must sum to one (Kacser and Burns 1973).

### 2.4 Toy protein allocation model illustration

**Figure 1.**
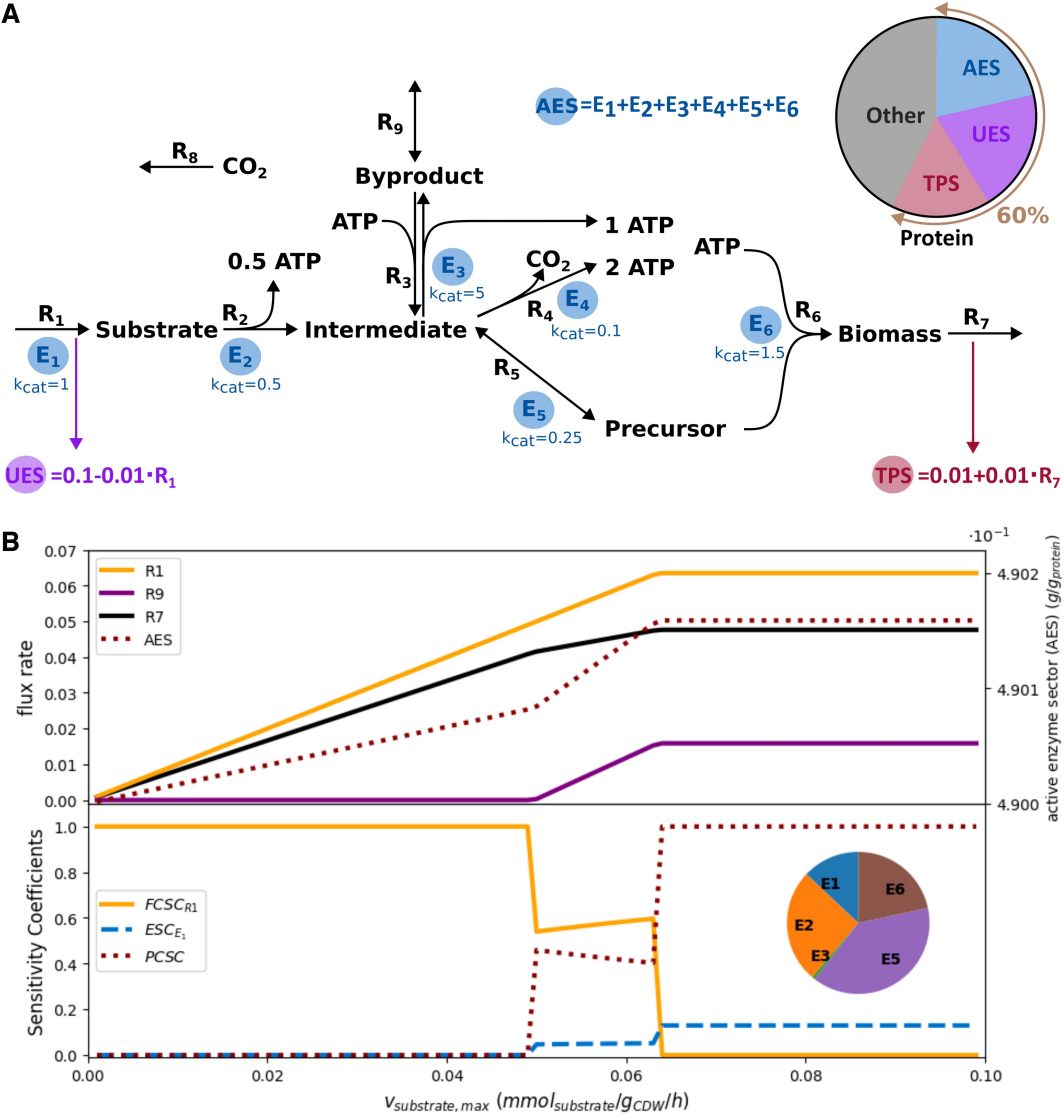
Toy protein allocation model (PAM) illustration of the changes in capacity and variable sensitivity coefficients. (A) Network representation of the toy model. *R* represent reaction identifiers, whereas *E* represent enzyme identifiers with their corresponding turnover values ($k_{\text{cat}}$). The active enzyme sector (AES), translational protein sector (TPS), and unused enzyme sector (UES) make up 60% of the total biomass dry weight. (B) Changes in flux rates, protein capacity, and sensitivity coefficients as a function of the maximal substrate consumption rate $v_{\text{substrate},\;\text{max}}$. $v_{\text{biomass}}$ increases linearly with $v_{\text{substrate},\text{max}}$ up to the point where the proteome becomes limiting as indicated by the nonzero proteome CSC (PCSC). The pie chart displays the distribution of other ESCs in the third phase of the simulation ($v_{\text{substrate},\text{max}}\geq 0.065\;\text{mmol}/g_{\text{CDW}}/h$) at full proteome control.

To introduce the capacity and enzyme sensitivity coefficients in an intuitive fashion, a simplified metabolic model is presented (Fig. 1A). The model includes reactions representing glycolysis (**R2**), the TCA cycle (**R4**, **R5**), and fermentation (**R3**) using simplified stoichiometries for illustration. There are two ATP generation routes: (i) an enzymatically inefficient pathway through R4 with higher ATP yield resembling respiration, and (ii) an enzymatically efficient pathway through R3 with lower ATP yield resembling fermentation (Fig. 1A). The model consists of three enzyme sectors: active enzymes (metabolic enzymes), translational protein, and unused enzymes. Based on the flux and proteome CSCs, three distinct phases can be identified for a range of substrate uptake rates. In the first phase, up to $v_{\text{substrate},\;\text{max}}\approx 0.05$ mmol/$g_{\text{CDW}}$/h, the flux CSC of **R1** limits the maximum specific biomass production rate ($v_{\text{biomass}}$)—here the objective function—for any given substrate uptake rate (Fig. 1B). Further increase in the biomass production rate in this regime relies on ATP from **R3**, producing byproducts. In the second phase ($0.050\leq v_{\text{substrate},\text{max}}\leq 0.065$ mmol/$g_{\text{CDW}}$/h), both the flux CSC of **R1** and the proteome CSC limit the biomass production rate. At this point, the ESC of enzyme 1, catalyzing the substrate uptake reaction, becomes nonzero and starts exerting control over $v_{\text{biomass}}$. In this phase, the biomass production rate can be improved by either increasing the total available protein or the substrate uptake rate. The third phase is characterized by a shift of the CSCs from flux to proteome capacity, indicating that all available proteome is optimally exploited for growth. When the total protein available for metabolic enzymes does not increase any further, the proteome becomes the only factor limiting biomass formation, and the proteome CSC becomes 1. As there is no proteome capacity for additional proteins, $v_{\text{biomass}}$ cannot increase further even if the substrate uptake is increased. The CSCs and ESCs indicate that growth can be improved by either making catabolic processes more protein efficient, or by increasing the available protein fraction.

## 3 Materials and methods

### 3.1 Models

PAMs are enzyme-constrained models with protein sectors representing metabolic, translational, and unused enzymes (Alter *et al.* 2021). The translational protein and unused enzyme sectors are included as linear equations (represented by $w^{T}v$ in (P2), line 4), which, in addition to the total concentration of metabolic enzymes (represented by $m^{T}e$ in (P2), line 4), constitutes the total available proteome (represented by $\phi_{0}$ in (P2), line 4). The parameters required to build the protein sectors for all models used in this work can be found in Supplementary Table S2.

### 3.2 *Escherichia coli* protein allocation models

To study the effect of model size on the computational performance of sensitivity calculations, a toy model (50 constraints, 30 variables), a core model (523 constraints, 320 variables), and a genome-scale model (12 818 constraints, 8182 variables) were employed. To build a core *E. coli* PAM, the *E. coli* core metabolic model built by Orth *et al.* (2010a) served as a template. The sectors were calibrated as described by Alter *et al.* (2021), using proteomics data from Schmidt *et al.* (2016) and known turnover numbers for enzymes present in the core model. The genome-scale *E. coli* PAM from Alter *et al.* (2021), based on the iML1515 metabolic model (Monk *et al.* 2017), was used with default parameters. An enzyme-constrained model was deduced from the PAM model by omitting the translational protein and unused enzyme sector while keeping the active enzyme sector. This model resembles the GECKO formulation (Sánchez *et al.* 2017), and was used for comparative purposes. All models contain a constraint limiting the sum of all proteins (Supplementary Table S2).

### 3.3 eGFP overexpression

Protein overexpression was modeled by adding the enhanced green fluorescent protein (eGFP) and the consumption of amino acids required to express the eGFP protein in the model. The amino acid sequence was taken from Bienick *et al.* (2014). Model predictions were validated against experimental data of an *E. coli* TUNER strain overexpressing eGFP (Bienick *et al.* 2014). In order to ensure consistency with the other simulations conducted as part of this study, the default total protein content was employed, although the *E. coli* TUNER strain was shown to have a higher total protein content than the wild-type *E. coli* strains (Alter *et al.* 2021). To prevent biological implausible byproduct formation under aerobic conditions as a consequence of the increased protein burden, the reaction converting pyruvate to formate, catalyzed by pyruvate formate lyase (PFL), was inactivated in the model. This adjustment agrees with the observation that the PFL complex in *E. coli* operates solely under anaerobic conditions (Boyington *et al.* 1997, Nnyepi *et al.* 2007).

### 3.4 Model simulations

To determine the computational performance of the sensitivity coefficients calculations, different PAMs were used and constrained to a substrate uptake rate of 9.82 mmol/$g_{\text{CDW}}/h$, the maximal computational glucose uptake rate reported for *E. coli* (Alter *et al.* 2021). The same substrate uptake rate was used to perform protein overexpression simulations. Similarly, toy PAM simulations were conducted by constraining the limiting nutrient exchange reaction and maximizing biomass formation (reaction **R7**, Fig. 1A). After each simulation, the CSCs and ESCs were calculated using the primal and dual variables obtained from the linear solver after the PAM resolution.

### 3.5 Numerical approximation of sensitivity coefficients

The computational efficiency and robustness of sEnz was evaluated against reported numerical methods for computing ESCs based on finite difference approximations. First-order finite difference approximations have previously been employed to estimate flux control coefficients (FCCs) in the ecYeast8 model (Lu *et al.* 2019). This method approximates the objective function sensitivity by a relative difference of the objective function value upon a marginal perturbation ($\Delta k_{\mathrm{cat}}$) in a catalytic constant value, e.g., $\Delta k_{\mathrm{cat}}=0.1\%$ of the nominal value. A more accurate approximation of the derivative was obtained using a central difference approximation. While the latter requires solving an additional optimization problem per sensitivity coefficient, it has a higher precision (second order) than the forward difference approximation (first order). We refer to these methods as numerical methods. Details about these approximations can be found in the Supplementary Text 1.3.

### 3.6 Implementation

All simulations were performed in Python 3.9 with the COBRApy v.0.29.0 (Ebrahim *et al.* 2013) and the PAModelpy v.0.0.3.3 toolboxes on an Ubuntu 22.04.3 LTS machine with 32 GB of RAM and an Intel i7-1265U ten-core processor at 4.00 GHz. The linear problems were solved with the GUROBI v.10.0 solver with default settings (Gurobi Optimization 2023). All scripts used in the analysis are available in Python and MATLAB on GitHub (https://github.com/iAMB-RWTH-Aachen/PAModelpy).

## 4 Results

### 4.1 Computational performance

To evaluate the performance of sEnz, the computational time required for the computation of sensitivity coefficients was compared to the calculation of so-called Flux Control Coefficients (FCCs) as calculated by Lu *et al.* (2019). In the latter case, two finite difference approximation schemes were employed, called forward and central (Supplementary Text 1.3). Both ESCs calculated with sEnz and FCCs quantify the sensitivity of the objective function value upon changes in enzyme variables, with ESCs representing precise values and FCCs providing numerical approximations. While the central difference approximation required roughly double the time compared to the forward scheme, its accuracy was superior, as indicated by the sum of sensitivity coefficients matching the exact ESCs in the medium-size model (Table 2). In all cases, the computational time required to calculate the sensitivity coefficients using sEnz was substantially lower. Compared to the fastest numerical method, sEnz was 28, 276 and more than 9300 times more efficient for the toy, core and genome-scale *E. coli* model, respectively. Notably, numerical computation of the sensitivity coefficients was simply impractical in the larger PAM (Table 2). On the contrary, determining the enzyme sensitivity coefficients for the genome-scale model using sEnz could be achieved within seconds, and, in the case of the core PAM, the numeric values of the coefficients yielded no notable differences as compared to the central difference approximation (Table 2, Supplementary Fig. S1). These results underscore the superior efficiency and robustness of the sensitivity calculations of sEnz when compared to traditional numerical schemes.

**Table 2.** Calculation of sensitivity coefficients using different methods in models of different sizes^a^

	Flux control coefficients				sEnz	
Model	Forward difference approximation^b^		Central difference approximation			
	Time (s)	∑ FCC	Time (s)	∑ FCC	Time (s)	∑ ESC
Toy PAM	0.533 ± 0.0118	1.000	1.020 ± 0.020	1.000	0.0401 ± 0.0009	1.000
*E. coli* core PAM	38.80 ± 2.015	0.697	76.80 ± 3.090	0.764	0.373 ± 0.018	0.764
iML1515 PAM	^c^	–	^c^	–	12.80 ± 0.711	0.711

a The computational time is given as the mean ± standard deviation of five replicate simulations.

b Numerical approximation implemented by Lu *et al.* (2019).

c Estimates based on the computational time of calculating a single coefficient were 20 and 57 h, respectively.

### 4.2 Phenotypical investigation of metabolic control

**Figure 2.**
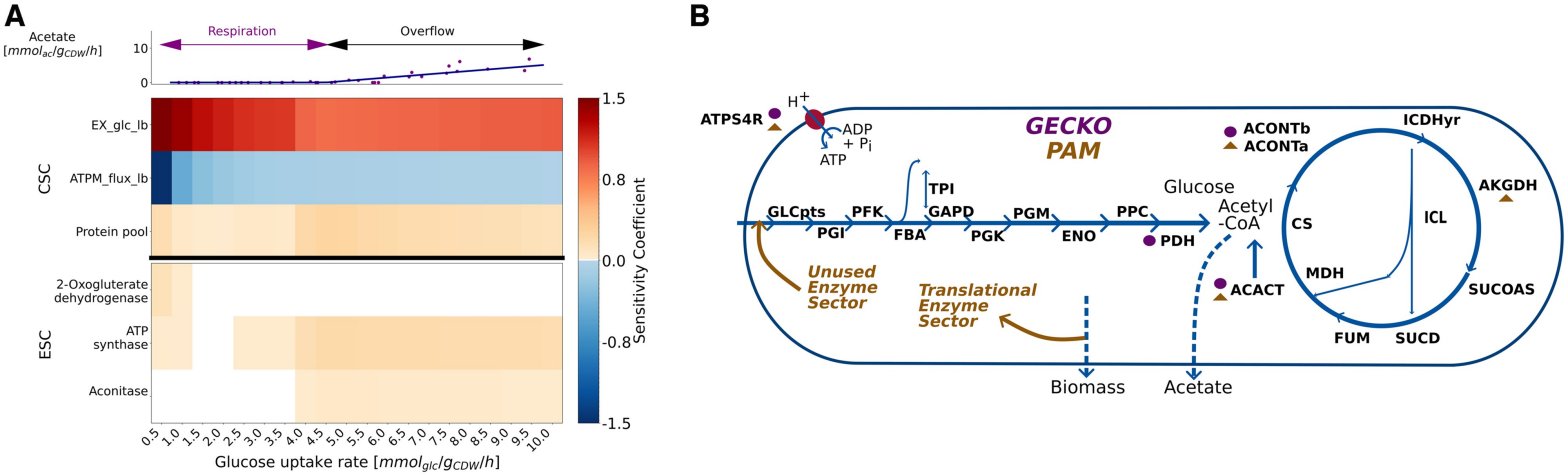
Overview of the main reactions and enzymes with the largest capacity sensitivity coefficients (CSC) and enzyme sensitivity coefficients (ESC) as a function of the glucose uptake rate in a genome-scale *E. coli* PAM (A). The top line graph shows the simulated acetate excretion rate, highlighting the predicted onset of overflow metabolism. The points represent experimental measurements from Schmidt *et al.* (2016). The arrows indicate the metabolic regime (respiration or overflow). EX_glc: glucose uptake reaction, ATPM: ATP maintenance, LB: lower bound. The CSCs include the proteome (protein pool) and flux CSCs. Only sensitivity coefficients with a magnitude >0.05 are shown. (B) Overview of the most sensitive enzymes in the genome-scale *E. coli* PAM and GECKO model (Supplementary Fig. S2). Squares indicate the top 5 enzymes in the GECKO-like model, and triangles indicate the top 5 enzymes in the PAM. The list of abbreviations can be found in Supplementary Table S3.

The proposed sensitivity coefficients, i.e. CSCs and ESCs, enable unraveling the intricacies of metabolism by estimating the control of reactions and enzymes on the objective function value. To study this relationship, sensitivity coefficients for a range of glucose uptake rates were calculated in a genome-scale *E. coli* PAM and a GECKO-like model incorporating only the active enzyme sector (Fig. 2; Supplementary Figs. S2 and S3). In both models, the glucose uptake and ATP maintenance reaction dominate the metabolic phenotype in the respiratory regime leading to intuitive metabolic findings. The positive value of the flux CSC for the glucose uptake implies that by increasing its value, the biomass production rate increases. Similarly, the negative flux CSC of the ATP maintenance reaction indicates that by decreasing the ATP maintenance burden, the specific biomass production rate increases.

In the overflow regime, the protein pool limits biomass production, resulting in acetate secretion, which is in line with previous findings (Beg *et al.* 2007, de Groot *et al.* 2020, Zeng and Yang 2020) (Fig. 2A). This effect is overestimated in the GECKO model in contrast to the PAM due to the presence of relations between protein sectors and glucose uptake (Supplementary Fig. S2). In the PAM, the unused enzyme sector negatively correlates with the substrate uptake rate, resulting in a decrease in protein burden with increasing glucose uptake rate (Fig. 2B). Higher glucose uptake results in an increased biomass production rate, which leads to an increased protein burden on the protein pool exerted by the translational protein sector (Alter *et al.* 2021).

By assessing the ESCs, which represent the contribution of enzymes to the biomass production, we can pinpoint key metabolic drivers and modulators. During overflow metabolism, when protein pool capacity constrains growth, enzymes with the highest ESCs control the growth rate. Following findings from MCA, enzymes with low efficiencies, e.g., low $k_{\text{cat}}$ to molecular weight ratios, or, high demand, exert more control on biomass production, correlating with higher sensitivity (Moreno-Sánchez *et al.* 2008). For the *E. coli* PAM, these are the pyruvate dehydrogenase complex (PDH), aconitase, and acetyl-coa acyltransferase (Fig. 2B). PDH is a protein complex with relatively high resource costs (Chen and Nielsen 2019), playing a central role communicating between glycolysis and the TCA cycle (Ogasawara *et al.* 2007). Aconitase and acetyl-CoA acyltransferase are amongst the least efficient proteins in the central carbon metabolism in this model (Supplementary Table S4). Aconitase has also been shown to be strongly affected by glucose dependent acetylation, highlighting its importance for the metabolization of glucose (Schilling *et al.* 2015). Overall, most control is exerted by enzymes associated with ATP generation, central carbon metabolism and biosynthesis (Supplementary Fig. S3). Another intriguing discovery utilizing ESCs is that, in parts of the respiratory regime (glucose uptake rate of 0.5 mmol/$g_{\text{CDW}}/h$), 2-oxoglutarate dehydrogenase imposes significant constraints on growth. Importantly, ESCs enable unraveling the limiting factors in different metabolic regimes.

### 4.3 Application example: metabolic control during protein overexpression

**Figure 3.**
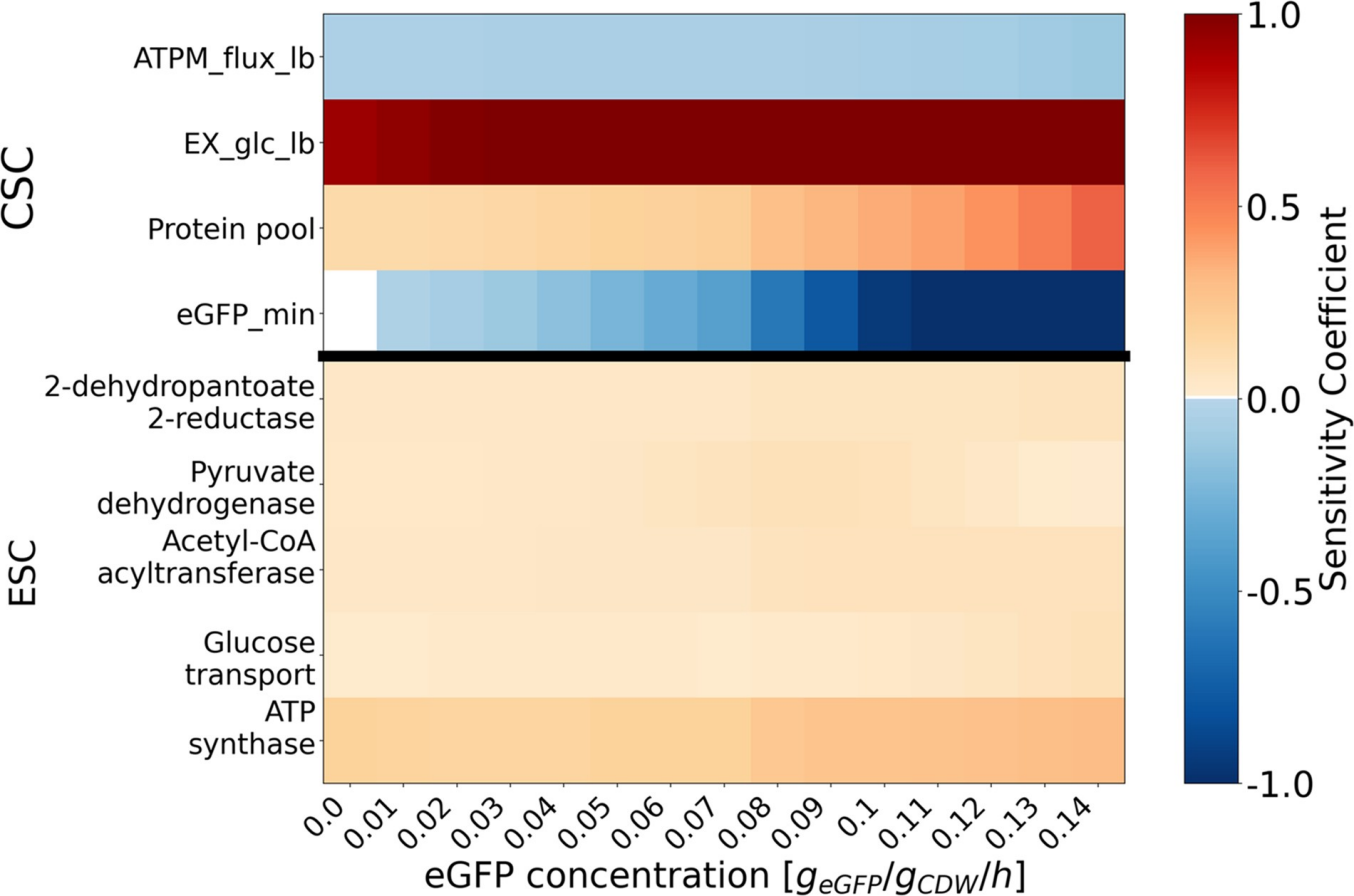
Overview of the key reactions and enzymes with the largest capacity sensitivity coefficients (CSC) and enzyme sensitivity coefficients (ESC) as a function of the concentration of the metabolically inactive enhanced green fluorescent protein (eGFP) in the genome-scale *E. coli* PAM. EX_glc: glucose uptake reaction, ATPM: ATP maintenance, LB: lower bound. The CSCs include the proteome CSC (protein pool) and flux CSCs. Only sensitivity coefficients ≥0.05 are shown.

To showcase how sEnz can improve the understanding of the metabolic phenotype for a genetically engineered strain, simulations of overexpression of eGFP were performed using the genome-scale *E. coli* PAM (Fig. 3). In the wild-type *E. coli* PAM, the protein pool and glucose uptake rate determine the metabolic phenotype. The ESCs indicate that the least efficient enzymes in the TCA cycle and respiratory pathway exert the highest control (refer to Section 4.2; Fig. 2). Upon overexpression of eGFP, which decreases the available fraction for other proteins, these sensitivities intensify (Fig. 3).

The influence of glucose uptake on the maximum biomass production rate increases with increasing protein burden. As previously explained, the glucose uptake rate is related to the unused enzyme sector and the translational sector in PAMs, and thus, it partially reflects the influence of the protein pool on simulated phenotypes. This result indicates that reducing the protein demand for catabolic processes can enhance growth at higher eGFP expression rates. Interestingly, with increasing protein burden, a shift from acetate to pyruvate excretion is observed. As a consequence the control of pyruvate dehydrogenase decreases (Fig. 3; Supplementary Fig. S4).

In all simulations of overflow metabolism, the ESC of ATP synthase indicates that this enzyme exerts the highest control over the protein pool constraint. The proteome CSC and glucose flux CSC indicate that changes in the total protein constraint will have the largest effect on the biomass production rate and thus metabolic phenotype (Fig. 3). Consequently, ATP synthase stands out as a prime candidate for metabolic engineering interventions for influencing growth performance. To investigate the impact of enhancing the efficiency of this enzyme, we conducted simulations wherein its turnover rate was doubled (Supplementary Fig. S5). Although the relation between the normalized growth rate and eGFP concentration was similar to the PAM without an ATP synthase perturbation (Supplementary Fig. S6), the simulations yielded a higher biomass production rate (Supplementary Fig. S4E). Furthermore, overflow metabolism and pyruvate production occur at a later stage, as indicated by a delayed onset of acetate excretion (Supplementary Fig. S4A). Upon decreasing the protein burden of ATP synthase, other enzymes increase their relative control (Supplementary Fig. S5). PDH and acyl-CoA acyltransferase emerged as enzymes with substantial control alongside ATP synthase. The former have important functions in metabolism by providing the components for acetyl-CoA and exhibit relatively low catalytic efficiency (Supplementary Table S4). In addition, the role of glucose uptake, and thus, the role of the enzyme sectors in the protein pool is decreased. These results illustrate how knowledge obtained from examining ESCs can guide improvements in microbial bioproduction.

## 5 Discussion

Studying the sensitivity of flux predictions can offer valuable information about the behavior of a metabolic network and its limitations. For this task, sEnz is an efficient and scalable method for the calculation of sensitivity coefficients in PAMs.

### 5.1 Computational scalability

Understanding the computational costs for sensitivity calculations is essential for assessing the scalability to models of different sizes. Calculation of sensitivity coefficients by sEnz was superior in both speed and accuracy compared to numerical methods (Table 2). The accuracy of the FCCs decreases with model size, while the computational time for the accurate central finite difference method explodes for genome-scale PAMs. Importantly, as there are no theoretical relations for the outcomes of numerical methods, it is challenging to assess their accuracy. In contrast, sEnz can exploit deviations from Equations (7), (11), and (12) to assess numerical accuracy.

### 5.2 Phenotypic control

The sensitivities obtained from sEnz give rise to a deeper understanding of the underlying biological mechanisms at play by highlighting reactions and enzymes exerting the highest control on the metabolic phenotype. For *E. coli* grown on a glucose minimal medium, glucose uptake and ATP maintenance expectedly limit growth in the respiratory regime, whereas the protein pool is the major limiting factor in the overflow regime. Previous studies employing simple kinetic models (Kovárová-Kovar and Egli 1998) have demonstrated the influence of substrate concentration on growth, and emphasized the impact of the protein pool on overflow (Beg *et al.* 2007, de Groot *et al.* 2020, Zeng and Yang 2020). The proteome CSC in the overflow regime indicates that a higher protein content leads to an increased biomass production. Indeed, in fast growing *E. coli* strains resulting from adaptive laboratory evolution, the majority of the differentially expressed genes were related to an increased capacity in protein synthesis (LaCroix *et al.* 2015). This observation corroborates the correlation between protein efficiency and growth rate, and underlines the applicability of sEnz for explorative studies and model curation: by examining the CSCs, we can identify and, if necessary, address the higher-level mechanisms governing the simulated metabolic phenotype.

The ESC values reveal enzyme-reaction constraints that contribute to protein burden and are potentially subject to selective pressure. Given that the $k_{\text{cat}}$ is the sole parameter in these constraints, ESCs also reflect $k_{\text{cat}}$ sensitivities. As a result, the numeric ESC values are strongly related to $k_{\text{cat}}$ values, thus the results of sEnz are dependent on the parametrization of the PAM. During overflow metabolism, enzymes involved in ATP generation, central carbon metabolism, and biosynthesis exhibit the highest ESCs (Supplementary Fig. S3), aligning with Bar-Even *et al.* (2011), who found that these enzymes experience significant selective pressure on their $k_{\text{cat}}$ values. This suggests that ESCs could not only serve as sensitivity measures, but also as signs of evolutionary selection pressure.

Among the enzymes associated to ATP generation, ATP synthase has the highest ESC, suggesting that it exerts the strongest control. ATP synthase is a bulky and essential protein in aerobic energy generation and has been demonstrated to be highly regulated in *E. coli* (Rabbers and Bruggeman 2022). Another study by Nilsson and Nielsen (2016) has shown a similar phenotype in yeast: ATP synthase is one of the main contributors to the protein pool, eventually limiting respiratory ATP generation. The observation that both ATP maintenance and ATP synthase exhibit growth-restricting characteristics underscores the significance of ATP availability in microbial growth (Chen and Nielsen 2019). The simulations of protein overexpression indicated that upon increasing the efficiency of ATP synthase, the protein burden decreases, resulting in higher biomass production rates and a delayed onset of overflow metabolism (Supplementary Fig. S5). Furthermore, the ESCs are able to pinpoint the least efficient enzymes in metabolic pathways. For example, in the *E. coli* PAM at a glucose uptake rate of 0.5 mmol/$g_{\text{CDW}}/h$, 2-oxoglutarate dehydrogenase contributes to limitation of biomass production, resulting in an unforeseen protein burden. This enzyme has been shown to play a limiting role in the respiratory regime, which coincides with its low catalytic efficiency (Tretter and Adam-Vizi 2005). Due to a relatively large fraction of unused protein at low biomass production rates (Alter *et al.* 2021), less resources are available for metabolic proteins, resulting in a higher protein burden exacerbated by this inefficient enzyme.

When overexpressing a metabolically inactive protein, protein reallocation is expected. Indeed, as a result of the increased protein burden, a shift in control from pyruvate dehydrogenase to other enzymes, such as Acetyl-CoA acyltransferase, can be observed as a result of pyruvate excretion (Supplementary Fig. S5). To this date, there are no data available to corroborate the predicted pyruvate excretion. Nevertheless, this result suggests that when the protein burden is increased, metabolism shifts to a more protein efficient energy generation pathway. More quantitative physiology data in protein overexpression conditions are required to validate this hypothesis.

### 5.3 Outlook

sEnz is a sound and efficient framework for investigating flux sensitivities in PAMs as function of metabolic capacities and enzyme concentrations. The proposed capacity and enzyme sensitivity coefficients are useful tools for understanding the metabolic limitations in an informative and simple manner. The sensitivity profiles visually show the influence of individual model parameters on metabolism, exemplified by the observable shift from purely respiratory metabolism to respiro-fermentation in glucose-limited *E. coli* cultivations. Although our results are intuitive, the profiles could reveal unforeseen patters for unconventional metabolic phenomena. Besides identifying key metabolic drivers, we envision sEnz as a useful tool for parametrizing PAMs. The ESCs can highlight the enzymes with the greatest influence on predicted metabolic phenotypes, thereby guiding parameter tuning. Furthermore, as exemplified by the simulations of protein overexpression and improved enzyme efficiency, sEnz holds promise for informing the design of targeted interventions for metabolic engineering tasks, where precise control of the metabolic phenotype is paramount. Our findings lay the groundwork for future investigations, encouraging a more holistic understanding of metabolic control under protein allocation constraints.

## Supplementary Material

btae691_Supplementary_Data

## Data Availability

The source code, along with all other Python scripts and notebooks used to reproduce the data generated for this publication, are available on GitHub at https://github.com/iAMB-RWTH-Aachen/PAModelpy.
