# Supplementary material for: Sensitivities in protein allocation models reveal distribution of metabolic capacity and flux control
Source: Bioinformatics. 2024 Nov 18;40(12):btae691. doi: 10.1093/bioinformatics/btae691 (PMC11631525; doi:10.1093/bioinformatics/btae691)
Supplement: btae691_Supplementary_Data [file btae691_supplementary_data.pdf]

Supplementary Information
Sensitivity of optimization-based flux predictions in protein allocation models reveals distribution of metabolic capacity and flux control

Samira van den Bogaard, Pedro A. Saa and Tobias B. Alter

November 7, 2024

Contents

1 Supplementary Text . . . **4**
1.1 Protein allocation model formulation and Dual formulation . . . 4
1.2 Relationships between primal and dual variables at the optimum . . . 5
1.3 Finite difference numerical approximations of parameter sensitivities . . . 6
1.3.1 Forward difference . . . 6
1.3.2 Central difference . . . 6
1.3.3 Sensitivity coefficient calculations . . . 6

2 Supplementary Figures . . . **7**

3 Supplementary Tables . . . **13**

List of Figures

S1 Distribution of sensitivity coefficients of the *E. coli* core PAM calculated with different methods. (**A**) Flux control coefficients (FCC) calculated with forward finite differences, (**B**) FCC calculated with central finite differences, (**C**) enzyme sensitivity coefficients calculated with sEnz. The x-axis shows the sensitivity coefficients on $\log_{10}$-scale. 7

S2 Overview of the main reactions or enzymes with the largest capacity sensitivity coefficients (CSC) and enzyme sensitivity coefficients (ESC) as a function of the glucose uptake rate for a full-scale *E. coli* GECKO protein allocation model (PAM) with only the active enzyme sector. The top line graph shows the simulated acetate excretion rate, which shows the predicted onset of overflow metabolism. The points represent experimental measurements from Schmidt et al. (2016). The arrows indicate the metabolic regime (respiration or overflow). EX_glc: glucose uptake reaction, ATPM: ATP maintenance, LB: lower bound. The CSCs include the proteome CSC (protein pool) and flux CSCs. Only sensitivity coefficients ≥ 0.05 are shown. 8

S3 Enzyme Sensitivity Coefficients (ESCs) mapped onto a proteomap of *E. coli*. The ESCs were calculated from simulations of the full-scale *E. coli* PAM with a glucose consumption rate of 10 mmol/g_{CDW}/h. **A** depicts the relative contribution of the overarching metabolic pathways and **B** the contribution of the individual enzymes to the protein burden. The reaction catalyzed by an enzyme is used as an intuitive identifier for the associated enzyme. The proteomaps were created using proteomaps.net (Bernhardt et al., 2009; Otto et al., 2010; Liebermeister et al., 2014). 9

S4 Simulated exchange rates as a function of eGFP expression. (**A**) Acetate production, (**B**) CO_2 production, (**C**) Oxygen consumption, (**D**) Pyruvate production and, (**E**) growth rate. Simulations were performed at a substrate uptake rate of 9.82 $\text{mmol}_{\text{glc}}/g_{\text{CDW}}/h$ 10

S5 Overview of the main reactions or enzymes with the largest capacity sensitivity coefficients (CSC) and enzyme sensitivity coefficients (ESC) as a function the concentration of an excess protein of the full-scale *E. coli* PAM where the enzymatic efficiency of ATP synthase is doubled. EX_glc: glucose uptake reaction, ATPM: ATP maintenance, LB: lower bound. The CSCs include the proteome CSC (protein pool) and flux CSCs. Only sensitivity coefficients ≥ 0.05 are shown. 11

S6 Normalized growth rate as a function of the eGFP concentration. The growth rate is normalized to the maximum growth rate without eGFP expression. The lines correspond to the simulations, the points to the experimental measurements with the error bars representing the variation as reported by Bienick et al. (2014). Simulations were performed at a substrate uptake rate of 9.82 $\text{mmol}_{\text{glc}}/g_{\text{CDW}}/h$. PAM: protein allocation model, $k_{\text{cat,ATPsynt}}$: k_{cat} of the ATP synthase enzyme complex. 12

List of Tables

S1 List of symbols and corresponding units 13

S2 Protein sector parameters for the models used in this study 14

S3 List of abbreviations of metabolic reactions as displayed in Figure 2B of the main text. The abbreviations originate from the iML1515 *E. coli* metabolic model (Monk et al., 2017). 14

S4 Enzyme efficiency in *E. coli* glycolysis and tricarboxylic cycle, related to protein allocation models used in this study. Efficiency is measured as grams of protein needed to convert 1 mol of substrate per hour. Enzyme identifiers correspond to the iML1515 model (Orth et al., 2010). 'f' denotes the forward reaction, 'b' the backward reaction. 15

1 Supplementary Text

Refer to Supplementary Table S1 for a description of each symbol used in the following derivations and the corresponding units.

1.1 Protein allocation model formulation and Dual formulation

The general protein allocation model (PAM) formulation can be written as follows (Alter et al., 2021),

$$
\begin{aligned}
\text{Primal PAM)}\ \max\ & v_z = \mathbf{c}^{\mathrm{T}}\mathbf{v} \\
\text{s.t.}\quad & \mathbf{S}\cdot\mathbf{v} = \mathbf{0} \\
& \mathbf{K}_{\mathrm{inv}}\cdot\mathbf{v} \leq \mathbf{e} \\
& \mathbf{w}^{\mathrm{T}}\mathbf{v} + \mathbf{m}^{\mathrm{T}}\mathbf{e} \leq \phi_0 \\
& \mathbf{v}_{\mathrm{min}} \leq \mathbf{v} \leq \mathbf{v}_{\mathrm{max}} \\
& \mathbf{e}_{\mathrm{min}} \leq \mathbf{e} \leq \mathbf{e}_{\mathrm{max}} \\
& v_i, e_j \in \mathbb{R}_0^+ \\
& i \in \{1,..,n\},\ j \in \{1,..,n_{\mathrm{enz}}\}
\end{aligned} \tag{1}
$$

This formulation is not in canonical form, which precludes formulation of its dual form. To this end, equality constraints must be recast as inequalities, which can be done by introducing two sets of inequality equations. In brief, let us consider the generic matrix linear equation $\mathbf{A}\cdot\mathbf{x} = \mathbf{b}$, then this equation can be expressed by two inequalities: i) $\mathbf{A}\cdot\mathbf{x} \leq \mathbf{b}$, and ii) $\mathbf{A}\cdot\mathbf{x} \geq \mathbf{b}$. Accordingly, the PAM formulation can be written in canonical form as follows,

$$
\begin{aligned}
\text{Primal PAM canonical)}\ \max\ & v_z = \mathbf{c}^{\mathrm{T}}\mathbf{v} \\
\text{s.t.}\quad & \mathbf{S}\cdot\mathbf{v} \leq \mathbf{0} \\
& -\mathbf{S}\cdot\mathbf{v} \leq \mathbf{0} \\
& \mathbf{K}_{\mathrm{inv}}\cdot\mathbf{v} - \mathbf{e} \leq \mathbf{0} \\
& \mathbf{w}^{\mathrm{T}}\mathbf{v} + \mathbf{m}^{\mathrm{T}}\mathbf{e} \leq \phi_0 \\
& \mathbf{v}_{\mathrm{min}} \leq \mathbf{v} \leq \mathbf{v}_{\mathrm{max}} \\
& \mathbf{e}_{\mathrm{min}} \leq \mathbf{e} \leq \mathbf{e}_{\mathrm{max}} \\
& v_i, e_j \in \mathbb{R}_0^+ \\
& i \in \{1,..,n\},\ j \in \{1,..,n_{\mathrm{enz}}\}
\end{aligned} \tag{2}
$$

Finally, for a general linear program (LP) written in canonical form:

$$
\begin{aligned}
\text{Primal LP)}\ \max\ & \mathbf{c}^{\mathrm{T}}\mathbf{x} \\
\text{s.t.}\quad & \mathbf{A}\cdot\mathbf{x} \leq \mathbf{b} \\
& \mathbf{x} \geq \mathbf{0}
\end{aligned} \tag{3}
$$

Its dual form is given by,

$$
\begin{aligned}
\text{Dual LP)}\ \min\ & \mathbf{b}^{\mathrm{T}}\mathbf{y} \\
\text{s.t.}\quad & \mathbf{A}^{\mathrm{T}}\cdot\mathbf{y} \geq \mathbf{c} \\
& \mathbf{y} \geq \mathbf{0}
\end{aligned} \tag{4}
$$

Application of the previous result (Dual LP) to PAM canonical yields the dual form for the formulation,

$$
\begin{aligned}
\text{Dual PAM)}\ \min\ & \mathbf{v}_{\max}^{\mathrm{T}}\boldsymbol{\mu}_{\max} - \mathbf{v}_{\min}^{\mathrm{T}}\boldsymbol{\mu}_{\min} + \mathbf{e}_{\max}^{\mathrm{T}}\boldsymbol{\varepsilon}_{\max} - \mathbf{e}_{\min}^{\mathrm{T}}\boldsymbol{\varepsilon}_{\min} + \phi_0\pi \\
\text{s.t.}\quad & \mathbf{S}^{\mathrm{T}}\boldsymbol{\lambda} + \mathbf{K}_{\mathrm{inv}}^{\mathrm{T}}\boldsymbol{\xi} + \boldsymbol{\mu}_{\max} - \boldsymbol{\mu}_{\min} + \pi\cdot\mathbf{w} \geq \mathbf{c} \\
& -\boldsymbol{\xi} + \boldsymbol{\varepsilon}_{\max} - \boldsymbol{\varepsilon}_{\min} + \pi\cdot\mathbf{m} \geq \mathbf{0} \\
& \pi, \xi_j, \mu_{\max,i}, \mu_{\min,i}, \varepsilon_{\max,j}, \varepsilon_{\min,j} \in \mathbb{R}_0^+;\ \lambda_i \in \mathbb{R} \\
& i \in \{1,..,n\},\ j \in \{1,..,n_{\mathrm{enz}}\}
\end{aligned}
\tag{5}
$$

1.2 Relationships between primal and dual variables at the optimum

If there exist feasible primal and dual solutions for Primal PAM) and Dual PAM), respectively, then by strong duality:

$$\bar{v}_z = \mathbf{v}_{\max}^{\mathrm{T}}\bar{\boldsymbol{\mu}}_{\max} - \mathbf{v}_{\min}^{\mathrm{T}}\bar{\boldsymbol{\mu}}_{\min} + \mathbf{e}_{\max}^{\mathrm{T}}\bar{\boldsymbol{\varepsilon}}_{\max} - \mathbf{e}_{\min}^{\mathrm{T}}\bar{\boldsymbol{\varepsilon}}_{\min} + \phi_0\bar{\pi} \tag{6}$$

where the bar denotes optimal solution variables. Eq. 6 can be normalized by $\bar{v}_z$ and written in the form of a summation using terms that resemble control coefficients. The resulting expressions are termed Capacity Sensitivity Coefficients (CSCs),

$$\sum_{i=1}^{n} C_{\mu,i}^{v_z} + \sum_{j=1}^{n_{\mathrm{enz}}} C_{\varepsilon,j}^{v_z} + C_{\pi}^{v_z} = 1 \tag{7}$$

Where,

$$
\begin{aligned}
C_{\mu,i}^{v_z} &= \frac{1}{\bar{v}_z}(v_{\max,i}\cdot\bar{\mu}_{\max,i} - v_{\min,i}\cdot\bar{\mu}_{\min,i}) \\
C_{\varepsilon,j}^{v_z} &= \frac{1}{\bar{v}_z}(e_{\max,j}\cdot\bar{\varepsilon}_{\max,j} - e_{\max,j}\cdot\bar{\varepsilon}_{\min,j}) \\
C_{\pi}^{v_z} &= \frac{\bar{e}_j}{\bar{v}_z}\bar{\pi}
\end{aligned}
\tag{8}
$$

An important result can be derived for the previous first two definitions. By definition, dual variables correspond to shadow prices at the optimum.

$$
\begin{aligned}
\bar{\mu}_{\max,i} &= \frac{\partial\bar{v}_z}{\partial v_{\max,i}} \approx \frac{\Delta\bar{v}_z}{\Delta v_{\max,i}} \\
\bar{\mu}_{\min,i} &= \frac{\partial\bar{v}_z}{\partial v_{\min,i}} \approx \frac{\Delta\bar{v}_z}{\Delta v_{\min,i}}
\end{aligned}
\tag{9}
$$

For $\Delta(\cdot) \approx 0$ such that the optimal basis does not change. If any of the primal inequalities is inactive, then the corresponding dual variable is zero. On the contrary, if the primal inequality is active, then the corresponding dual variable is nonzero. In the latter case, there is a one-to-one correspondence between the constraint bound ($v_{\max,i}$ or $v_{\min,i}$) and the reaction flux (v_i), i.e., $v_{\max,i} = \bar{v}_i$ and/or $v_{\min,i} = \bar{v}_i$ whenever the primal constraint is active. Then, we can rewrite the definition for the corresponding CSC as follows provided we are at the optimum,

$$C_{\mu,i}^{v_z} = \frac{\bar{v}_i}{\bar{v}_z}(\bar{\mu}_{\max,i} - \bar{\mu}_{\min,i}) = \frac{\bar{v}_i}{\bar{v}_z}\Delta\bar{\mu}_i \tag{10}$$

The above result can be extended for the sensitivity coefficient $C_{e,j}^{v_z}$, yielding the following result:

$$C_{\varepsilon,j}^{v_z} = \frac{\bar{e}_j}{\bar{v}_z}\Delta\bar{\varepsilon}_j \tag{11}$$

1.3 Finite difference numerical approximations of parameter sensitivities

For the numeric approximation of parameter sensitivities in protein allocation models, the finite difference calculations as described below were used. As these calculations were only applied on enzyme variables, with $k_{\text{cat}} > 0$, the case in which $\theta^* = 0$ is not described.

1.3.1 Forward difference

$$\left.\frac{\partial f}{\partial \theta}\right|_{\theta^*} \approx \frac{f(\theta^* + \Delta\theta) - f(\theta^*)}{\Delta\theta} \tag{12}$$

Where typically $\Delta\theta = 0.01\theta^*$ (i.e., perturbation corresponds to 1% of the nominal parameter value represented by θ^*). The approximation error for $\left.\frac{\partial f}{\partial \theta}\right|_{\theta^*}$ is of the order of $\sim O(\Delta\theta)$

1.3.2 Central difference

$$\left.\frac{\partial f}{\partial \theta}\right|_{\theta^*} \approx \frac{f(\theta^* + \Delta\theta) - f(\theta^* - \Delta\theta)}{2\Delta\theta} \tag{13}$$

Where typically $\Delta\theta = 0.01\theta^*$ (i.e., perturbation corresponds to 1% of the nominal parameter value represented by θ^*). The approximation error for $\left.\frac{\partial f}{\partial \theta}\right|_{\theta^*}$ is of the order of $\sim O(\Delta\theta^2)$

1.3.3 Sensitivity coefficient calculations

Both numerical methods were employed to estimate the relevant sensitivity coefficients by normalizing the absolute sensitivity value by the ratio of the nominal values of the function f and parameter θ^*.

$$\text{Relative sensitivity} = \frac{\theta^*}{f(\theta^*)} \left.\frac{\partial f}{\partial \theta}\right|_{\theta^*} = \left.\frac{\partial \ln f}{\partial \ln \theta}\right|_{\theta^*} \tag{14}$$

Flux control coefficients (FCC) reported by (Lu et al., 2019) correspond to the above expression using a forward approximation for the absolute sensitivity. In this particular case, $v_z^* = f(\theta^*)$ and $\theta^* = k_{\text{cat},j}$ so the relative sensitivity describes a FCC.

2 Supplementary Figures

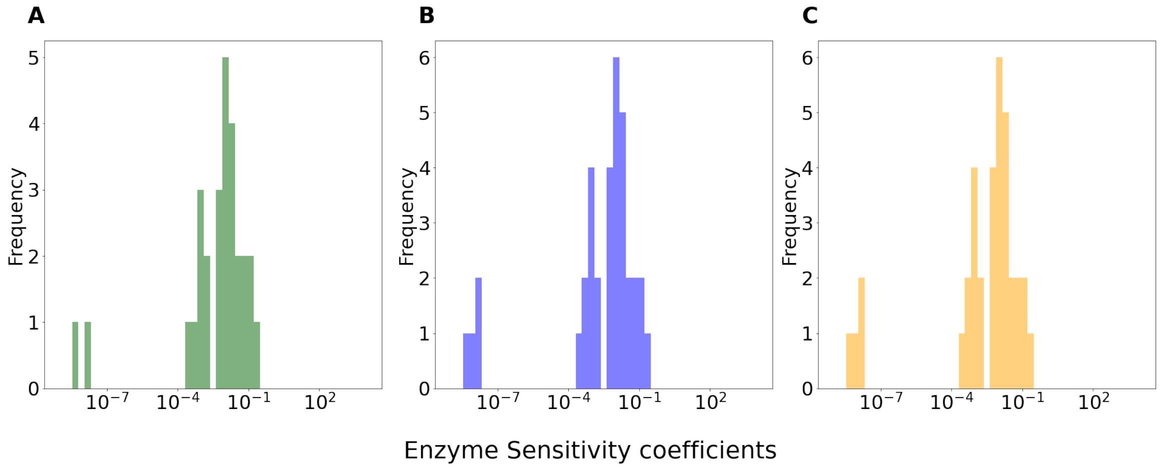

Figure S1: Distribution of sensitivity coefficients of the *E. coli* core PAM calculated with different methods. (**A**) Flux control coefficients (FCC) calculated with forward finite differences, (**B**) FCC calculated with central finite differences, (**C**) enzyme sensitivity coefficients calculated with sEnz. The x-axis shows the sensitivity coefficients on $\log_{10}$-scale.

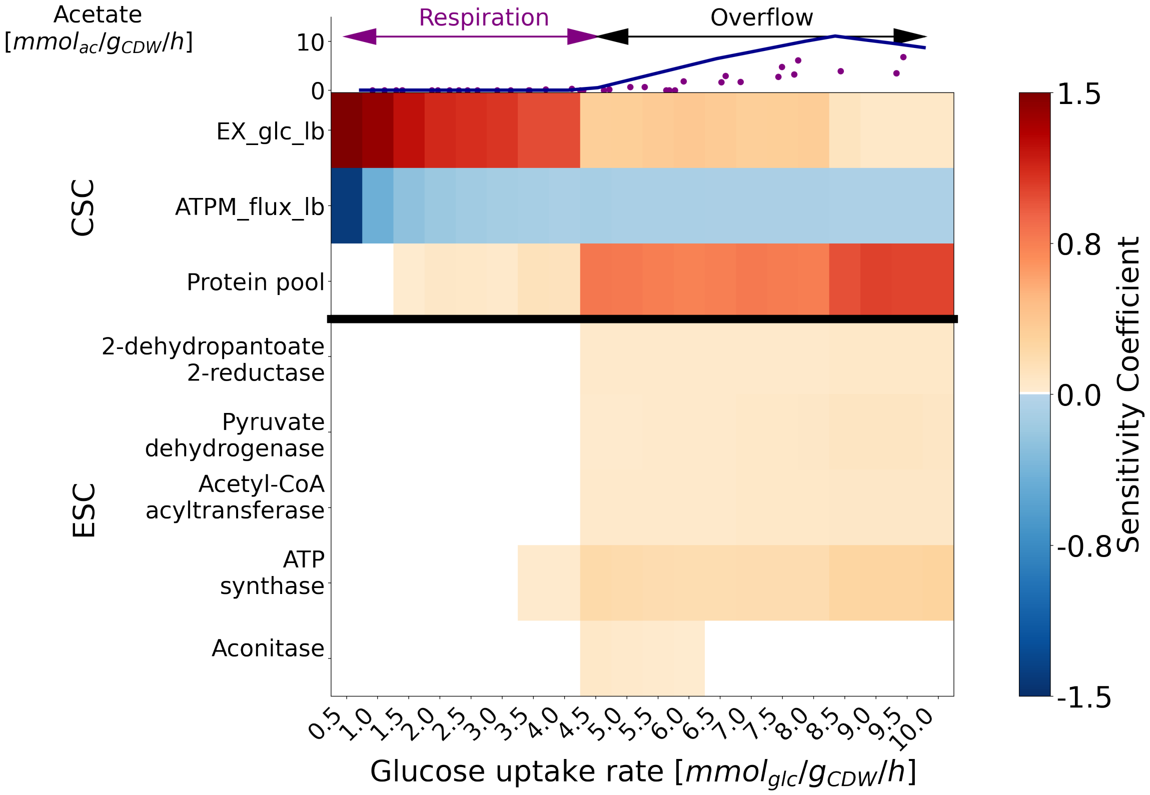

Figure S2: Overview of the main reactions or enzymes with the largest capacity sensitivity coefficients (CSC) and enzyme sensitivity coefficients (ESC) as a function of the glucose uptake rate for a full-scale *E. coli* GECKO protein allocation model (PAM) with only the active enzyme sector. The top line graph shows the simulated acetate excretion rate, which shows the predicted onset of overflow metabolism. The points represent experimental measurements from Schmidt et al. (2016). The arrows indicate the metabolic regime (respiration or overflow). EX_glc: glucose uptake reaction, ATPM: ATP maintenance, LB: lower bound. The CSCs include the proteome CSC (protein pool) and flux CSCs. Only sensitivity coefficients ≥ 0.05 are shown.

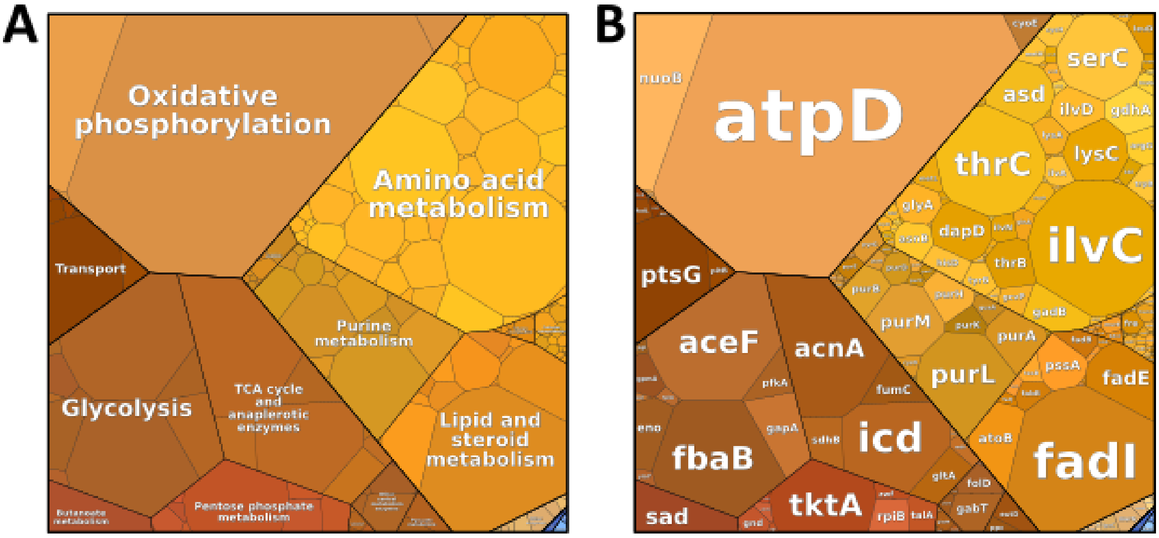

Figure S3: Enzyme Sensitivity Coefficients (ESCs) mapped onto a proteomap of *E. coli.* The ESCs were calculated from simulations of the full-scale *E. coli* PAM with a glucose consumption rate of 10 mmol/g_{CDW}/h. **A** depicts the relative contribution of the overarching metabolic pathways and **B** the contribution of the individual enzymes to the protein burden. The reaction catalyzed by an enzyme is used as an intuitive identifier for the associated enzyme. The proteomaps were created using proteomaps.net (Bernhardt et al., 2009; Otto et al., 2010; Liebermeister et al., 2014).

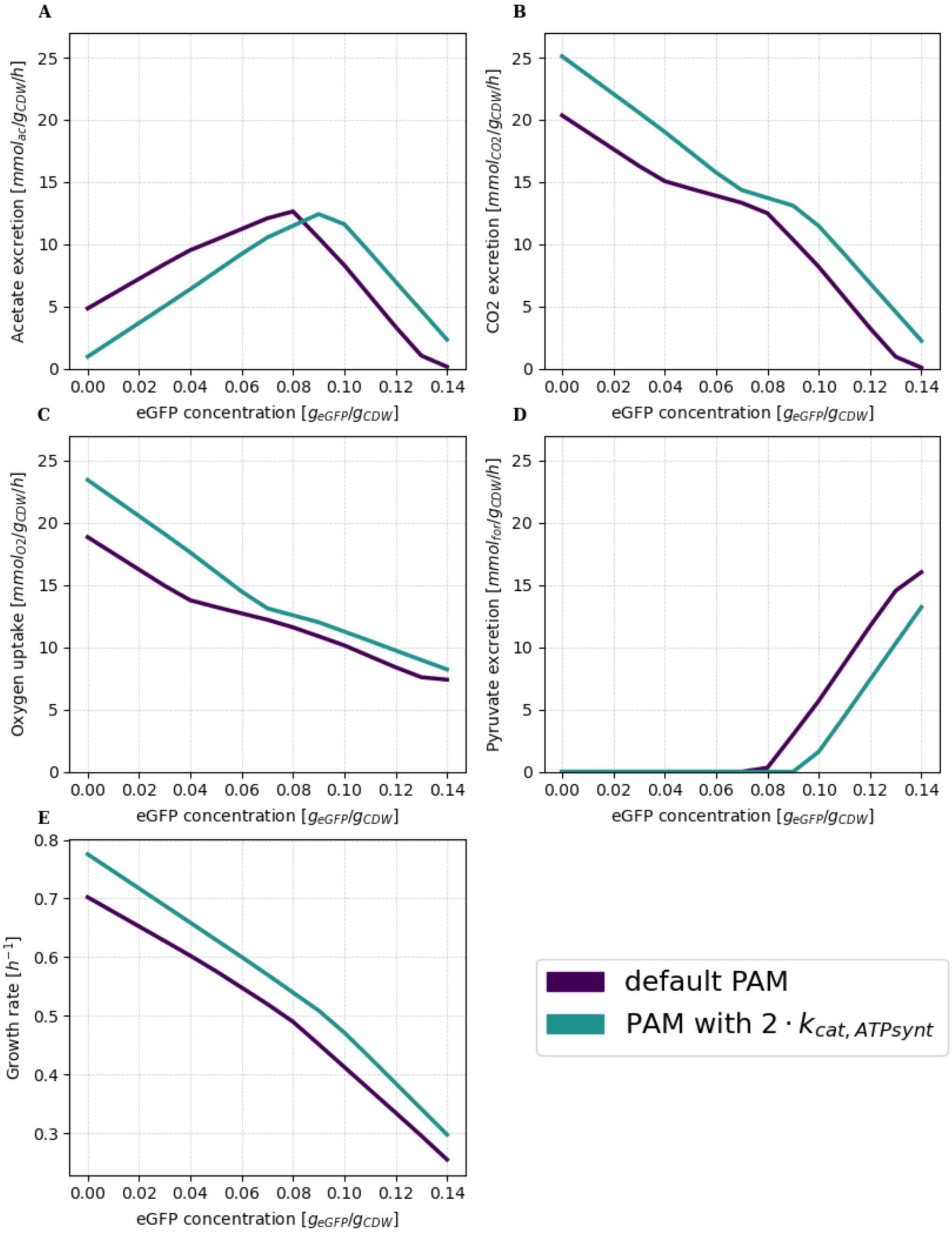

Figure S4: Simulated exchange rates as a function of eGFP expression. (**A**) Acetate production, (**B**) CO_2 production, (**C**) Oxygen consumption, (**D**) Pyruvate production and, (**E**) growth rate. Simulations were performed at a substrate uptake rate of 9.82 $\mathrm{mmol_{glc}/g_{CDW}}/h$

.

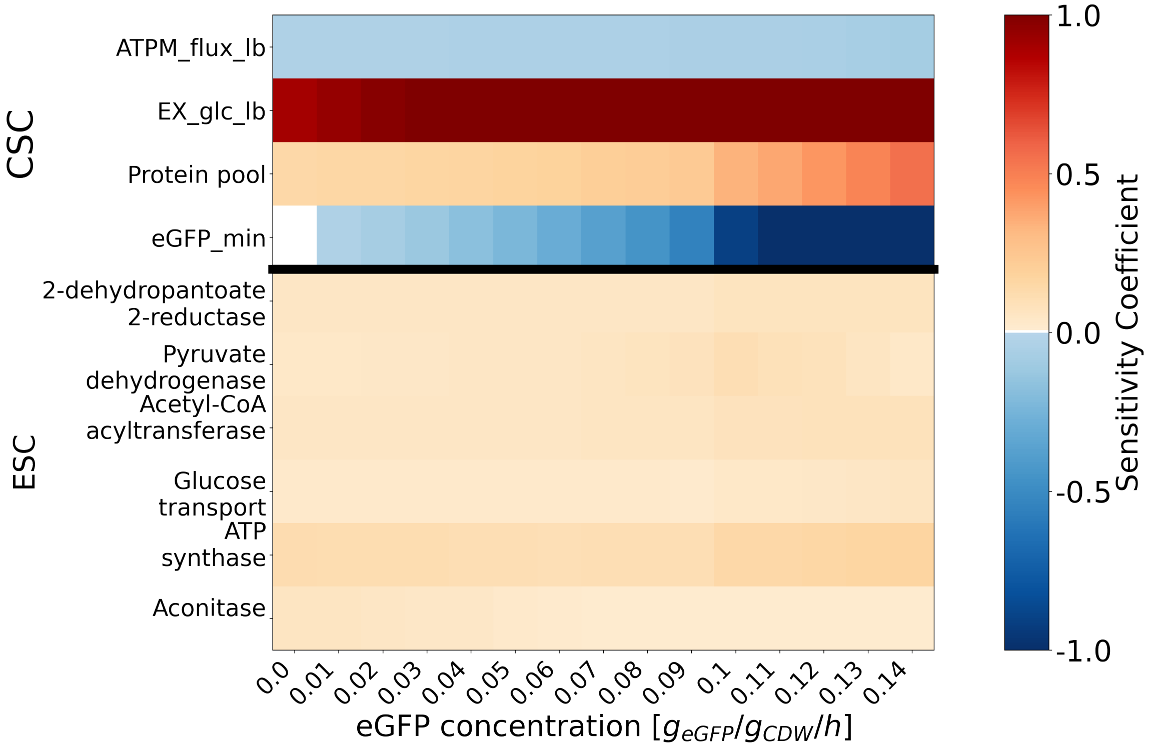

Figure S5: Overview of the main reactions or enzymes with the largest capacity sensitivity coefficients (CSC) and enzyme sensitivity coefficients (ESC) as a function the concentration of an excess protein of the full-scale *E. coli* PAM where the enzymatic efficiency of ATP synthase is doubled. EX_glc: glucose uptake reaction, ATPM: ATP maintenance, LB: lower bound. The CSCs include the proteome CSC (protein pool) and flux CSCs. Only sensitivity coefficients ≥ 0.05 are shown.

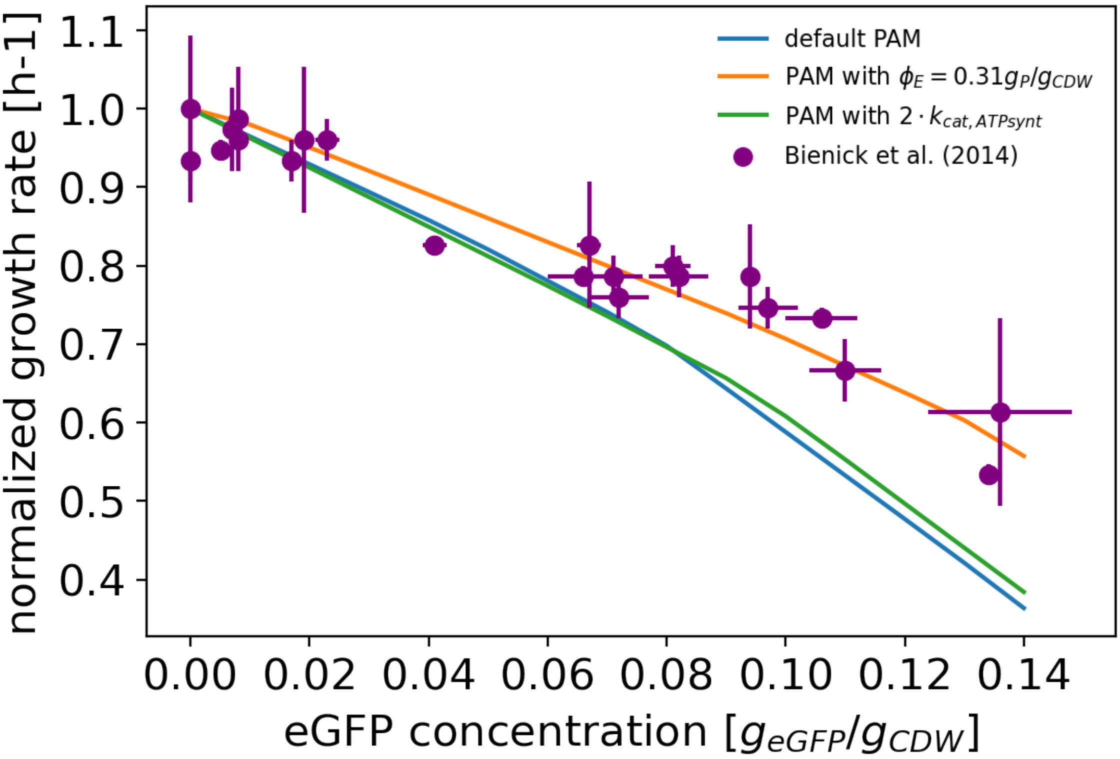

Figure S6: Normalized growth rate as a function of the eGFP concentration. The growth rate is normalized to the maximum growth rate without eGFP expression. The lines correspond to the simulations, the points to the experimental measurements with the error bars representing the variation as reported by Bienick et al. (2014). Simulations were performed at a substrate uptake rate of 9.82 $\text{mmol}_{\text{glc}}/\text{g}_{\text{CDW}}/h$. PAM: protein allocation model, $k_{\text{cat,ATPsynt}}$: k_{cat} of the ATP synthase enzyme complex.

3 Supplementary Tables

Table S1: List of symbols and corresponding units

	Symbol	Unit	Description
Primal variables	$\mathbf{S}$	-	Stoichiometric matrix
	$\mathbf{c}$	-	objective vector
	n	-	Number of biochemical reactions
	n_{enz}	-	Number of metabolic enzymes
	m	-	Number of metabolites
	v	$\text{mmol/g}_{\text{CDW}}/h$	Reaction rate
	v_{min}	$\text{mmol/g}_{\text{CDW}}/h$	Lower bound of reaction
	v_{max}	$\text{mmol/g}_{\text{CDW}}/h$	Upper bound of reaction
	v_z	$\text{mmol/g}_{\text{CDW}}/h$	Objective reaction flux
	k_{cat}	$1/h$	Catalytic turnover rate
	e	$\text{mmol/g}_{\text{CDW}}$	Enzyme concentration
	e_{min}	$\text{mmol/g}_{\text{CDW}}$	Minimal enzyme abundance
	e_{max}	$\text{mmol/g}_{\text{CDW}}$	Maximal enzyme abundance
	ϕ_0	$\text{mg}_{\text{protein}}/\text{g}_{\text{CDW}}$	Net total available proteome
	$\mathbf{m}$	g/mol	Vector of molar masses for the metabolic enzymes
	$\mathbf{K}_{\text{inv}}$	h	Diagonal matrix consisting of the inverse k_{cat} values of all enzyme-reaction associations
Dual variables	λ	-	Dual variable associated to a mass balance
	ξ	-	Dual variable associated to an enzyme constraint
	μ_{min}	-	Dual variable associated to the lower bound of a reaction
	μ_{max}	-	Dual variable associated to the upper bound of a reaction
	ε_{min}	-	Dual variable associated to the minimum abundance of an enzyme
	ε_{max}	-	Dual variable associated to the maximal abundance of an enzyme
	π	-	Dual variable associated to the total protein content
Sensitivity coefficients	α	$\text{g}_{\text{AES}}/\text{g}_{\text{totprot}}$	Fraction of the total proteome (totprot) allocated to the active enzymes sector (AES)
	$C_{\mu}^{v_z}$	-	Flux Capacity Sensitivity Coefficient (FCSC)
	$C_{\varepsilon}^{v_z}$	-	Enzyme Capacity Sensitivity Coefficient (ECSC)
	$C_{\pi}^{v_z}$	-	Proteome Capacity Sensitivity Coefficient (PCSC)
	$C_{e}^{v_z}$	-	Enzyme Sensitivity Coefficient (ESC)

Table S2: Protein sector parameters for the models used in this study

Model	Unused Enzyme Sector		Translational Enzyme Sector		Total Protein
	$\phi_{UE,0}$ g_p/g_{CDW}	w_{UE} $g_p/mmol_{glc}/h$	$\phi_{T,0}$ g_p/g_{CDW}	w_T $g_p/g_{CDW}/h$	ϕ_P g_p/g_{CDW}
Toy model	0.100	-0.010	0.010	0.100	0.450
E. coli core model	$4.07 \cdot 10^{-2}$	$-2.14 \cdot 10^{-2}$	$4.99 \cdot 10^{-2}$	$2.94 \cdot 10^{-3}$	0.170
E. coli PAM*	0.171	$-1.74 \cdot 10^{-2}$	$4.99 \cdot 10^{-2}$	$3.68 \cdot 10^{-3}$	0.258

$\star$ As described by Alter et al. (2021)

Table S3: List of abbreviations of metabolic reactions as displayed in Figure 2B of the main text. The abbreviations originate from the iML1515 *E. coli* metabolic model (Monk et al., 2017).

Abbreviation	Full reaction name
GLCpts	Glucose transport
PGI	Glucose-6-phosphate isomerase
PFK	Phosphofructokinase
FBA	Fructose bisphosphate aldolase
TPI	Triose-phosphate isomerase
GAPD	Glyceraldehyde-3-phosphate dehydrogenase
PGK	Phosphoglycerate kinase
PGM	Phosphoglycerate mutase
ENO	Enolase
PPC	Phosphoenolpyruvate carboxylase
CS	Citrate synthetase
ACONT	Aconitase
ICDHyr	Isocitrate dehydrogenase
AKGDH	Alpha-ketogluterate dehydrogenase
SUCOAS	Succinyl-CoA syntethase
SUCD	Succinate dehydrogenase
FUM	Fumerase
MDH	Malate dehydrogenase
ATPS4R	ATP synthase
PDH	Pyruvate dehydrogenase
ACACT	Acetyl-CoA C-acyltransferase
ICL	Isocitrate lyase

Table S4: Enzyme efficiency in *E. coli* glycolysis and tricarboxylic cycle, related to protein allocation models used in this study. Efficiency is measured as grams of protein needed to convert 1 mol of substrate per hour. Enzyme identifiers correspond to the iML1515 model (Orth et al., 2010). 'f' denotes the forward reaction, 'b' the backward reaction.

Enzyme	Molar mass $[\frac{g}{\text{mol}}]$	kcat $[h^{-1}]$	Enzymatic turnover efficiency $[g_{\text{protein}}/\text{mol}_{\text{substrate}}/h]$
AKGDH	199700.0	4320	46.23
ACACT_f	43700.0	5040	8.67
ACONTa_b	95580.0	18720	5.11
FBA_b	38630.0	9360	4.13
PGI_b	61530.0	18000	3.42
ACONTa_f	95580.0	70560	1.35
ICDHyr_f	45760.0	39960	1.15
FBA_f	38630.0	38880	0.99
PDH	216400.0	239220	0.90
PGM_f	42370.0	50400	0.84
ATPS4rpp_f	234000.0	284400	0.82
ACACT_b	43700.0	53280	0.82
SUCOAS_f	71150.0	94356	0.75
GLCptspp	151800.0	265680	0.57
ENO_b	45650.0	122652	0.37
SUCDi	118300.0	396000	0.30
FUM_f	56960.0	216000	0.26
CS	48010.0	183600	0.26
ACt2rpp	20070.0	79200	0.25
ACACT1r_b	42580.0	170640	0.25
ATPS4rpp_b	234000.0	1026000	0.23
NADH16pp	540200.0	2763000	0.20
ICDHyr_b	45760.0	317160	0.14
GAPD	35530.0	252000	0.14
PTAr_f	56620.0	432000	0.13
FUM_b	56960.0	464400	0.12
PFK	33650.0	287280	0.12
PYK	51040.0	487800	0.10
SUCOAS_b	71150.0	723600	0.10
ENO_f	45650.0	509040	0.09
NADTRHD	51560.0	604440	0.08
MDH_f	32340.0	498600	0.06
PGM_b	42370.0	990000	0.04
PTAr_b	56620.0	1494000	0.04
TPI_f	26970.0	1177200	0.02
PGI_f	61530.0	3325500	0.02
ACKr_f	43030.0	3268800	0.01
MDH_b	32340.0	3988800	$8.1 \cdot 10^{-3}$
PGK_f	41120.0	5328000	$7.7 \cdot 10^{-3}$
PGK_b	41120.0	9478800	$4.3 \cdot 10^{-3}$
ACKr_b	43030.0	12807000	$3.3 \cdot 10^{-4}$
TPI_b	26970.0	32400000	$8.3 \cdot 10^{-4}$

References

Alter, T. B., Blank, L. M., and Ebert, B. E. (2021). Proteome regulation patterns determine *Escherichia* coli wild-type and mutant phenotypes. *mSystems*, 6(2):e00625–20.

Bernhardt, J., Funke, S., Hecker, M., and Siebourg, J. (2009). Visualizing Gene Expression Data via Voronoi Treemaps. In *2009 Sixth International Symposium on Voronoi Diagrams*, pages 233–241.

Bienick, M. S., Young, K. W., Klesmith, J. R., Detwiler, E. E., Tomek, K. J., and Whitehead, T. A. (2014). The interrelationship between promoter strength, gene expression, and growth rate. *PLOS ONE*, 9(10):e109105. Publisher: Public Library of Science.

Liebermeister, W., Noor, E., Flamholz, A., Davidi, D., Bernhardt, J., and Milo, R. (2014). Visual account of protein investment in cellular functions. *Proceedings of the National Academy of Sciences*, 111(23):8488–8493. Publisher: Proceedings of the National Academy of Sciences.

Lu, H., Li, F., Sánchez, B., Zhu, Z., Li, G., Domenzain, I., Marcišauskas, S., Anton, P., Lappa, D., Lieven, C., Beber, M., Sonnenschein, N., E.J., K., and Nielsen, J. (2019). A consensus *S. cerevisiae* metabolic model yeast8 and its ecosystem for comprehensively probing cellular metabolism. *Nature Communications*, 10(1):3586.

Monk, J. M., Lloyd, C. J., Brunk, E., Mih, N., Sastry, A., King, Z., Takeuchi, R., Nomura, W., Zhang, Z., Mori, H., Feist, A. M., and Palsson, B. O. (2017). iML1515, a knowledgebase that computes *Escherichia coli* traits. *Nature biotechnology*, 35(10):904–908.

Orth, J. D., Fleming, R., and Palsson, B. (2010). Reconstruction and use of microbial metabolic networks: the core *Escherichia coli* metabolic model as an educational guide. *EcoSal Plus*, 4(1):10.1128/ecosalplus.10.2.1. Publisher: American Society for Microbiology.

Otto, A., Bernhardt, J., Meyer, H., Schaffer, M., Herbst, F.-A., Siebourg, J., Mäder, U., Lalk, M., Hecker, M., and Becher, D. (2010). Systems-wide temporal proteomic profiling in glucose-starved Bacillus subtilis. *Nature Communications*, 1(1):137. Publisher: Nature Publishing Group.

Schmidt, A., Kochanowski, K., Vedelaar, S., Ahrné, E., Volkmer, B., Callipo, L., Knoops, K., Bauer, M., Aebersold, R., and Heinemann, M. (2016). The quantitative and condition-dependent *Escherichia coli* proteome. *Nature Biotechnology*, 34(1):104–110.
